# Structural Layers of Ex Vivo Rat Hippocampus at 7T MRI

**DOI:** 10.1371/journal.pone.0076135

**Published:** 2013-09-26

**Authors:** Jeanine Manuella Kamsu, Jean-Marc Constans, Franck Lamberton, Patrick Courtheoux, Pierre Denise, Bruno Philoxene, Maelle Coquemont, Stephane Besnard

**Affiliations:** 1 Unité Mixte de Recherche, UMR 1075, Université de Caen, Caen, France; 2 Service de Radiologie, Centre Hospitalier Universitaire D’Amiens, Amiens, France; 3 Unité Mixte de Recherche, UMR 6194 Centre National de Recherche Scientiﬁque (CNRS), Commissariat à l’energie atomique (CEA), Université de Caen et Paris2, Paris, France; 4 Unité Imagerie par Résonance Magnétique Pôle Imagerie, Centre Hospitalier Universitaire Côte de Nacre, Caen, France; 5 Service d’histologie, Hôpital Côte de Nacre, Centre Hospitalier Universitaire Côte de Nacre, Université de Caen, Caen, France; Institute of Psychology, Chinese Academy of Sciences, China

## Abstract

Magnetic resonance imaging (MRI) applied to the hippocampus is challenging in studies of the neurophysiology of memory and the physiopathology of numerous diseases such as epilepsy, Alzheimer’s disease, ischemia, and depression. The hippocampus is a well-delineated cerebral structure with a multi-layered organization. Imaging of hippocampus layers is limited to a few studies and requires high magnetic field and gradient strength. We performed one conventional MRI sequence on a 7T MRI in order to visualize and to delineate the multi-layered hippocampal structure ex vivo in rat brains. We optimized a volumic three-dimensional T2 Rapid Acquisition Relaxation Enhancement (RARE) sequence and quantified the volume of the hippocampus and one of its thinnest layers, the stratum granulare of the dentate gyrus. Additionally, we tested passive staining by gadolinium with the aim of decreasing the acquisition time and increasing image contrast. Using appropriated settings, six discrete layers were differentiated within the hippocampus in rats. In the hippocampus proper or Ammon’s Horn (AH): the stratum oriens, the stratum pyramidale of, the stratum radiatum, and the stratum lacunosum moleculare of the CA1 were differentiated. In the dentate gyrus: the stratum moleculare and the stratum granulare layer were seen distinctly. Passive staining of one brain with gadolinium decreased the acquisition time by four and improved the differentiation between the layers. A conventional sequence optimized on a 7T MRI with a standard receiver surface coil will allow us to study structural layers (signal and volume) of hippocampus in various rat models of neuropathology (anxiety, epilepsia, neurodegeneration).

## Introduction

The hippocampal formation is a complex cerebral structure involved in episodic memory and spatial memory processes [1,2]. This multilayered structure is classically composed of the hippocampus proper and its CA1, CA2, and CA3 subfields, the dentate gyrus and the subiculum [3]. Magnetic Resonance Imaging (MRI) applied to the hippocampus is challenging in studies on the neurophysiology of memory and the physiopathology of numerous diseases such as epilepsy [4,5], ischemia [6], Alzheimer’s disease [7], and However, the types of damage observed by MRI remain nonspecific and poorly delineated, particularly in rodent brains. Regardless of the pathology, MRI findings are restricted to atrophy measurement and non-specific high signal intensity within the hippocampus on T2 or FLAIR sequence. Given that specific regions or layers of the hippocampus are affected early in the development of some pathologies, such as the CA1 region with Alzheimer’s disease [7], or the granular layer of the dentate gyrus with depression [8], it has become imperative to obtain more precise images of the hippocampal structure in order to improve our understanding and the early detection of microstructural changes which could occur in the hippocampus [9]. This can be achieved through images with high spatial resolution which allow us to distinguish hippocampal structural layers.

Compared to histology, immunohistochemistry and quantitative autoradiography procedures, conventional MRI sequences fail to distinguish hippocampal layers due to poor spatial resolution and signal-to-noise ratio (SNR) [4,10,11]. Only a few studies have reported MRI hippocampal layers in rodent [12,13,14,15]. However, they used higher field strength and complex processing methods. Working on a standard, widely used 7T MRI scanner, we performed one conventional sequence on ex vivo rat brains in order to visibly demonstrate the multilayer hippocampal structure and calculate their left hippocampal volumes and the volume of the stratum granulare within the dentate gyrus. We optimized a three-dimensional T2 Rapid Acquisition Relaxation Enhancement (3D RARE) sequence by varying numerous MRI parameters. We also quantified the volume of the hippocampus by MRI segmentation. The number of distinguishable layers was qualitatively compared to those obtained by a common histological staining procedure. Additionally, we report the principal effect of a passive staining procedure on the acquisition time and image parameters such as signal and contrast-to-noise ratios.

## Materials and Methods

### General procedures

Experiments were conducted in accordance with The European Community Council Directive 86/6609/EEC, as well as French legislation. The protocol was approved by our regional ethical committee (Comité d’Ethique Normandie en Matière d’Expérimentation Animale, CENOXEMA, number assigned 0412-01). Six male Sprague Dawley rats (300-400 g, Janvier, France) were housed in groups of 2-3 under constant temperature (21 ± 1°C), humidity (55 ± 5%) and lighting conditions (less than 110 Lux). Rats were kept under a 12: 12 h normal light: dark cycle (lights on at 8:00 a.m.) with food and water available *ad libitum*. One rat brain among the six had the passive staining procedure by contrast agent done before MRI. Then, MRI volumes and granule cell layer volumes of the unstained hippocampi were calculated and histological procedures were performed.

### Brain preparation for MRI processing

Rats were deeply anesthetized with an intraperitoneal injection of urethane (150 mg/kg). Each animal was then intracardially perfused with 200 mL of phosphate-buffered saline (PBS; 0.1 M, pH 7.4) followed by 200 mL of 4% paraformaldehyde in phosphate buffer (DiaPath). Brains were carefully removed by craniotomy, post fixed in 4% paraformaldehyde in phosphate buffer for 2 hours, and then stored at 4°C in PBS to be used for MRI acquisitions with or without gadolinium.

### MRI data acquisition

The brains were directly placed into a Falcon tube such that the anterior-posterior axis of the brain was collinear with the long axis of the tube. The tube was gradually warmed to room temperature over a 24-h period. The stock solution of PBS decreased the paraformaldehyde concentration of neural tissue by counter-diffusion and thus prevented T2 shortening as reported in fixed tissue samples [16]. Additionally, gently shaking and inverting the tube manually removed remaining small air bubbles. A small quantity of PBS was then added if necessary.

### MRI system

Acquisitions were performed on a Bruker Pharmascan 7-Tesla horizontal magnet (Ettlingen, Germany) with a 16-cm horizontal-bore magnet and a 9-cm (inner diameter) shielded gradient, A 1H resonance frequency of 300 MHz, a maximum gradient strength of 300 mT/m and a 200 ms rise time were used. The 20-mm receiver surface coil enabled an increase in SNR. Data acquisition and image processing was controlled by Paravision 5.1 Bruker software.

### MRI acquisition

Two short MRI series were acquired in order to define three mutually orthogonal planes (transversal, horizontal, sagittal) and to correct positioning of the editor box on the brain from coronal and sagittal images (15 slices). The coronal slice package was positioned perpendicular to the hemispheric fissure and the sagittal slice package parallel to the brainstem. Next, we tested four sets of parameters of 3D T2 RARE sequence in order to reveal the maximum number of hippocampal layers, including the granular cell layer.

### MRI parameters

**Figure 1:**
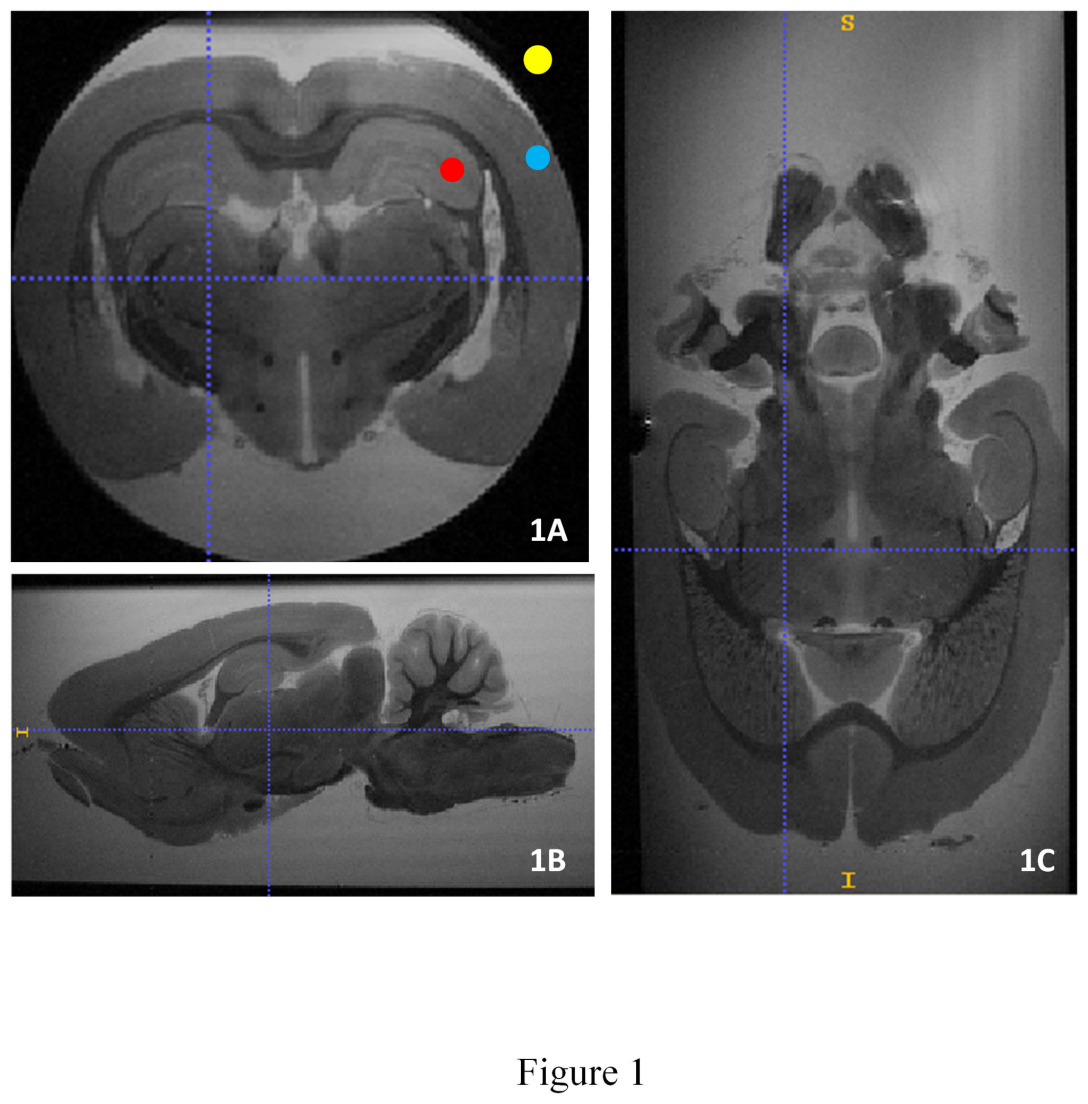
Regions of interest and 3D MRI acquisition. Positioning of ROI for the calculation of SNR and three-plane view of rat hippocampus. Blue: ROI placed on the cortex, red: ROI placed on the hippocampus, yellow: ROI placed on the background of the image for the noise calculation. a: coronal view, 1B: sagital view, 1C: axial view.

Four sets of operator-dependent parameters (the first was A1 and the fourth A4) were tested (Table 1) by varying spatial resolution parameters (FOV, matrix size, slice thickness), contrast parameters (TE, TR), and acquisition time parameters (TR, RARE factor, Nex) on one rat brain. Then, the best set of parameters (Table 1; A3 in bold) was chosen, taking into account criteria of image quality such as signal to noise ratio (SNR) and contrast-to-noise ratio (CNR). The sampling of the k-space line was linear with the RARE factor and the matrix size used. No fat saturation was applied because ex vivo brains had been stored in Phosphate Buffer Solution (PBS) and there was good contrast between PBS and cerebral tissue. The SNR was then calculated as the mean magnitude of the regions of interest (ROI) located within the dorsal part of hippocampus divided by the standard deviation of the noise (mean magnitude of an ROI located outside of the brain on the MRI image). The CNR was calculated as the difference in the signal between two ROIs located, respectively, in the granular layer and the cortex divided by the standard deviation of the noise (ITK SNAP 2.2) (Figure 1A). The best set of chosen parameters was then applied in the other rats.

**Table 1:** MRI parameters tested during whole brain acquisition.

		**A1**	**A2**	**A3**	**A4**
**Spatial Resolution**	FOV	4.6 x 2.3 x 2.3	4.6 x 1.8 x 1.8	4 x 1.6 x 1.6	3 x 1.5 x 1.5
	Size of matrix	512 x 250 x 256	512 x 200 x 200	512 x 200 x 200	512 x 256 x 256
	Partial k-space (y-direction)	-	-	-	0.77
	Zero-filling (z-direction)	-	-	-	0.72
	**Voxel size**	**90 µm3**	**90 µm3**	**80 µm3**	**60 µm3**
Contrast parameters	TR (ms)	2200	*2200*	1700	1900
	TE (ms)	93	*70*	63	71
**AT**		**11h 44 min**	**12h 06min**	**12h 28 min**	**12h 25 min**
	Rare Factor	10	6	6	6
	Nex	3	3	4	4

Different sets of MRI parameters (A1 to A4) tested on one unstained rat brain. In bold, the set of parameters chosen for our study.

### Contrast agent preparation and MRI sequence

One rat brain was incubated in a solution of PBS mixed with gadolinium (0.2 mL Dotarem in 10mL PBS) for 70 minutes just before MRI acquisition. Dose and time of incubation were adapted from [17]. Additionally, a RARE T1 MAP sequence was acquired in order to calculate the T1 relaxation times and to estimate the TR. Then, acquisition was carried out on the stained rat brain with the same 3D T2 RARE sequence parameters applied except for the TR.

### Image post processing

After transferring DICOM files, the hippocampi were segmented in a rostral to caudal direction according to Paxinos and Watson [18] on approximately 75 consecutive coronal slices. Hippocampal volume and the granule cell layer volume were then calculated using ITK-SNAP 2.2 software [19].

### Brain preparation for qualitative histology

After removal, four brains were stored in a 30% sucrose solution for histology. Brains were cut into 20-µm coronal sections on a cryostat at -24°C and mounted on slides (Ultra frost, Thermo, Fisher). Slides were treated with cresyl violet stain and observed under a microscope with an analog color camera (Zeiss Aviovert 135 and Axovision 4.4, Sony CCD Camera). The MRI layers were then qualitatively compared with the cresyl violet staining.

## Results

### MRI parameters

Our chosen 3D RARE sequence parameters offered the best compromise between spatial resolution, SNR and CNR, and acquisition time: TR/Effective TE = 1700ms/63 ms with four averages, a RARE Factor of 6, a total scan time of 12 h 30 min, a field of view at 4 x 1.6 x 1.6 cm, and a matrix of 512 x 200 x 200 with a spatial resolution of 80 x 80 x 80 µm^3^ (Table 1). The mean SNR and CNR of the different acquisitions, calculated for five rats, were respectively, 42± 8 and 5.5±0.77 (Table 2).

**Table 2:** MRI contrast agent parameters.

	A3 G70	A3 (unstained)
**TR (msec)**	450	1700
**AT (H)**	3	12.5
**T1 (msec)**	304±4.5	1440
**SNR**	41.8	42±8
**CNR**	15.6	5.5±0.77

Comparison of sets of parameters of the unstained or stained acquisition.

AG70 is the set of parameters obtained after 70 minutes of passive staining. SNR and CNR in A3 (unstained) are averages of data from 5 unstained rat brains.

### Visual demonstration of hippocampal layers

**Figure 2:**
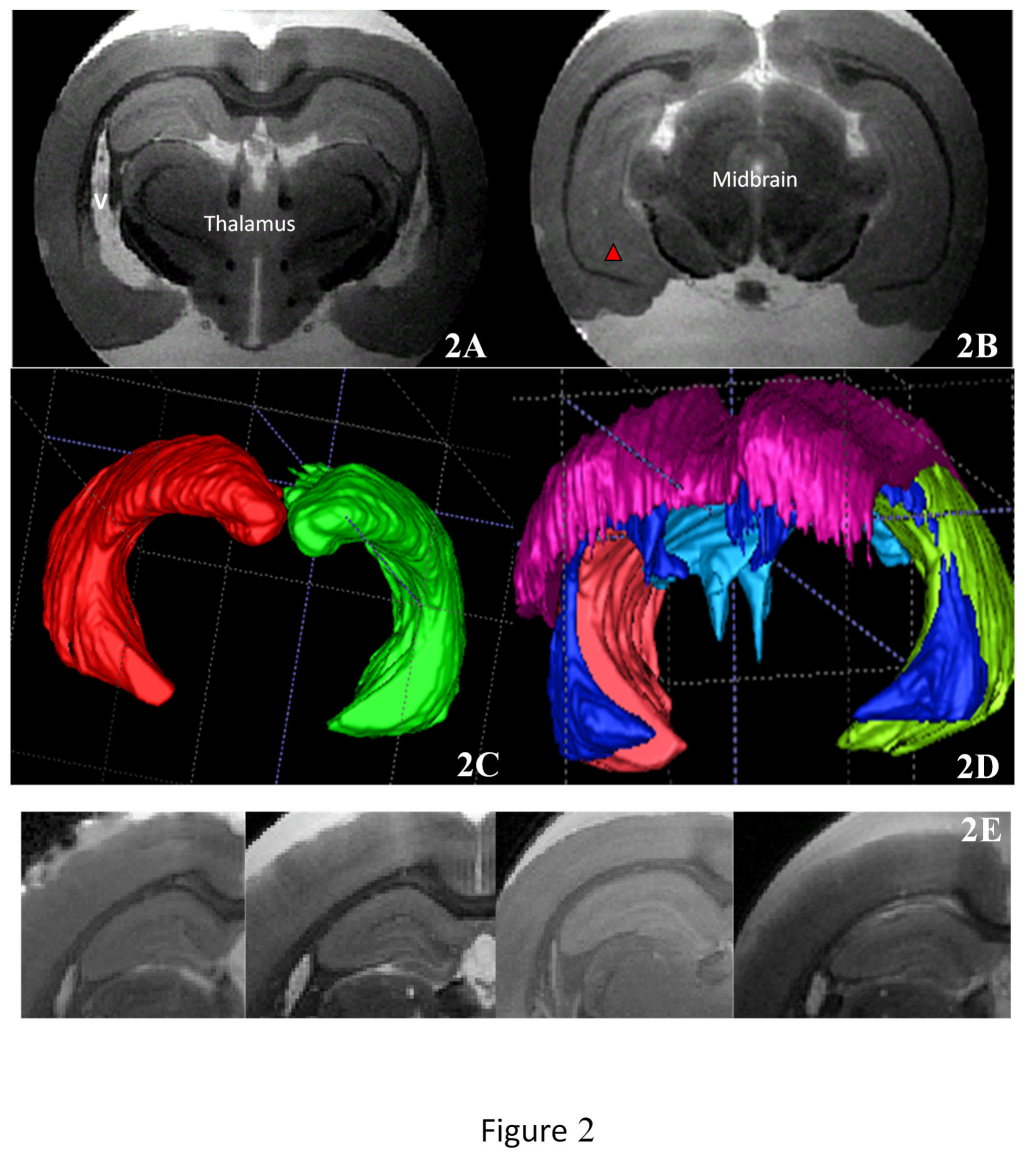
2A: 3D hippocampus and other brain structures imaging. 2A and 2B: limits of the hippocampus with adjacent structures. 2C: three-dimensional view of the hippocampus. Red: right hippocampus, green: left hippocampus. 2D: results of the segmentation of the other structures (Red: corpus callosum, blue: lateral ventricles, sky blue: fourth ventricle). 2E: Hippocampus layers obtained by our four sets of parameters from A1 (left) to A4 (right). The better visualisation of the six hippocampus layers is obtained with our A3 sets of parameters.

**Figure 3:**
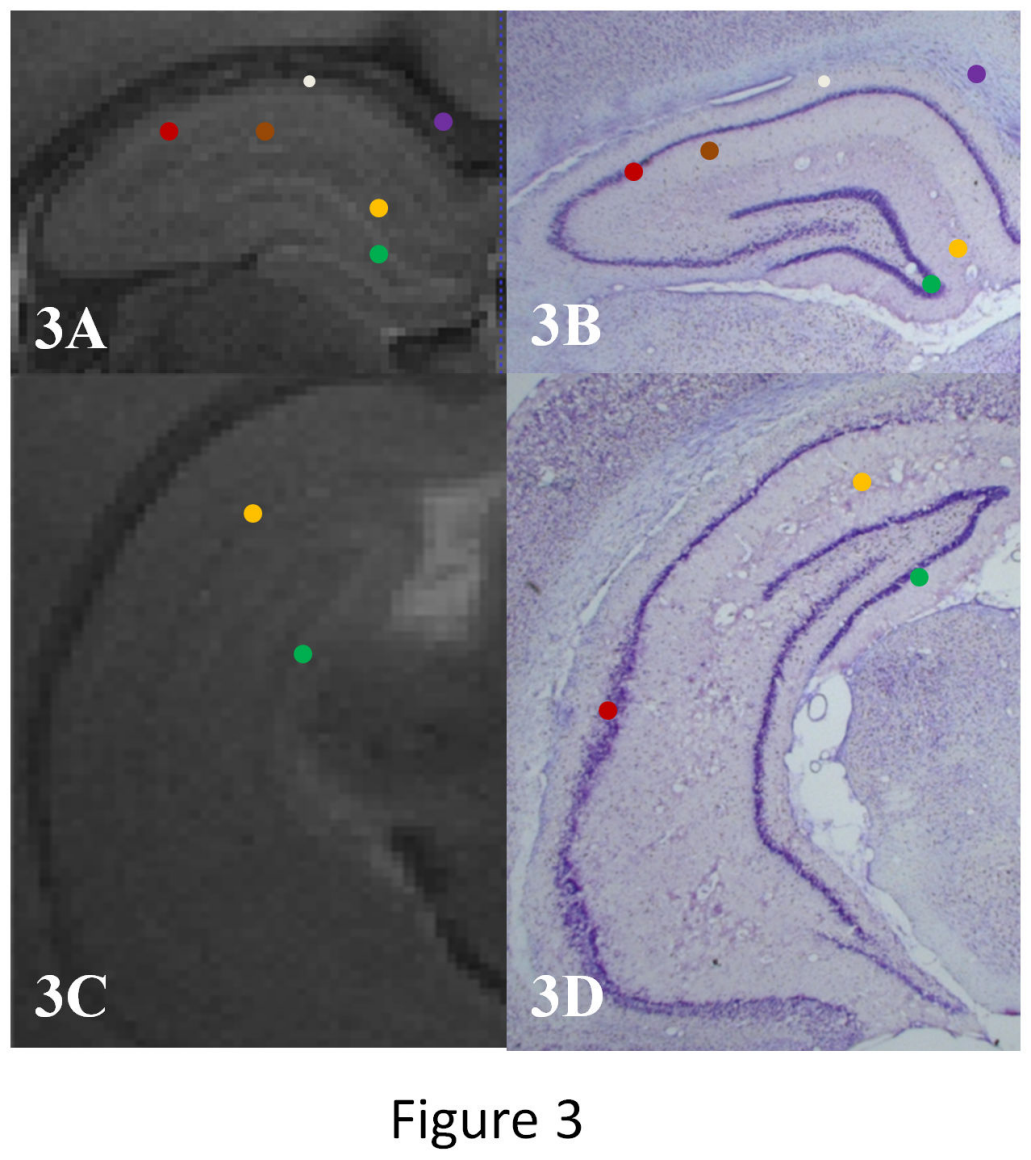
MRI and histological hippocampal sub-layers comparison. Comparison of T2-weighted images of the dorsal hippocampus (A) and ventral hippocampus (C) and cresyl violet’s histological slices (B and D). White dot: stratum oriens, Red: CA3 stratum pyramidale, Brown: stratum radiatum , Yellow: CA1 stratum lacunosum and DG stratum moleculare ,Blue: stratum moleculare of the dentate gyrus, Green: stratum granulare of the dentate gyrus, Purple: Corpus callosum.

**Figure 4:**
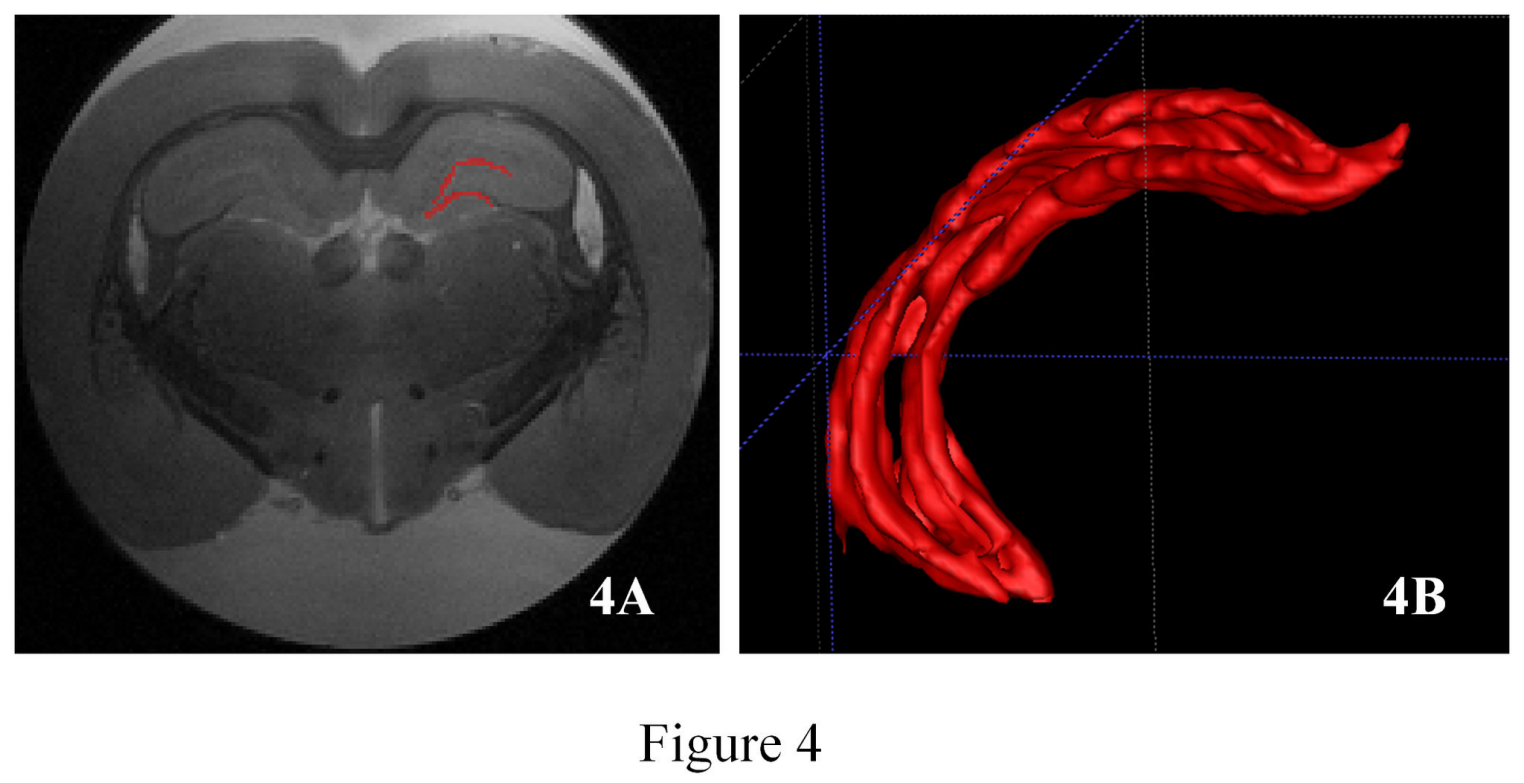
3D representation of stratum granular. Segmentation and volume rendering of the stratum granulare of the dentate gyrus. 4A Segmentation of stratum granulare on dorsal hippocampus, 4B Volume rendering of stratum granulare of the dentate gyrus.

Our MRI sequence allowed excellent contrast between cerebral structures. The signal of PBS solution appeared hyper-intense, contrasting with the iso-intense signal of the neural tissue (Figure 1). The hippocampus formation was clearly distinguishable from adjacent structures; the corpus callosum, located just above and the thalamus below appeared hypo-intense on the 3D T2 RARE sequence. Lateral ventricles were hyper- intense, allowing us to clearly distinguish the hippocampus (Figure 2A and 2B). A three-dimensional view resulting from the accumulation of 75±10 consecutive sections (Figure 2C) allowed us to clearly distinguish between the hippocampus and the other adjacent structures (Figure 2D). The ventral portion of the hippocampus was also seen to a lesser degree (Figure 2B, red arrow). Six different layers including the granule cell layer could also be visualized using a comparison with histological sections (Figure 3). The hippocampal formation comprises distinct structures: the dentate gyrus, the hippocampus proper with its three fields, CA1 to CA3, and the subiculum. From the dorsal to ventral portion of the hippocampus proper: the stratum oriens, the CA1 and CA3, stratum pyramidale, the stratum radiatum and the stratum lacunosum moleculare were distinctly seen. From the dorsal to the ventral portion of the dentate gyrus: the stratum moleculare and the stratum granulare were clearly distinguishable. It was also possible to obtain a three-dimensional view of the stratum granulare of the DG (Figure 4). These six layers were less distinct in the ventral part of the hippocampus. The stratum granulare and the stratum pyramidal appeared hyper-intense compared to the cortex. The stratum oriens, the stratum lacunosum moleculare and the stratum moleculare the stratum radiatum appeared iso-intense.

### Volume measurements

The mean volume of the five left hippocampi (Table 3), calculated from Magnetic Resonance Imaging, was 54±3.12 mm^3^. MRI hippocampus total volumes were calculated from 75±10 (SD) slices ranging from -1.72 to -8.04 mm from the Bregma, according to stereotaxic coordinates. To calculate the volume of the very thin stratum granulare of the dentate gyrus, we chose the acquisitions having the highest SNR and CNR (n=4) in order to optimize the measurements. The volume of the granular cell layer, calculated on four chosen rats from 56±3 MRI slices, was 1.4± 0.2 mm^3^ (Table 4).

**Table 3:** Review of MRI parameters.

		**Wolf et al. 2002**	**Benveniste et al. 2000/2002**	**Kalisch et al. 2006**	**Badea et al. 2007 Sharief 2008**	**Johnson et al. 2007**	**Besnard et al. 2012**	**Our study 013**	
Animal	Species	Rat Sprague-Dawley	Mice C57BL/6J	Rat Wistar	Mice C57BL/6J	Mice C57BL/6J	Rat Sprague-Dawley	Rat Sprague-Dawley	
	Age	9 weeks	Adult	12 weeks	9 weeks	9-12 weeks	12 weeks	12 weeks	
	Gender	Male		Male	NA	NA	Male	Male	
	Weight(g)	351±15	25-30	351±11	NA	NA	250-360	300-400	
**B0/Weighting**		**7T/T2SE**	9,4T/T^2^*	**7T/T^2^ SE RARE**	**9,4 T/3D multi écho T2**	**9,4T/3DSET2**	**7T/T^2^ RARE**	**7T/3DT2 RARE**	
MRI Parameters	TR (msec)	4000	150	4000	400	400	4000	1700
	TE (msec)	25	9	44	7-112(8 échoes)	56	16,8	63	
	Flip Angle		25	NA					
	Voxel size (µm3)	125x125 x600	39x39 x156	68 x68 x750	43x43 x43	43x43 x43	75x75 x700	80x80 x80	
	AT (hours)	2	NA	0.7	4.15	4,1	0.5	12.5	
**Volume (mm^3^)**		**96.33 (THV**)** i.e. 46.17 per H**	**NA**	**49.42±0.71(LHV**)	**NA**	**NA**	**58.1±3.3(LHV**)	**54± 3.12 (LHV**)	
**Number of layers**		**0**	**6**	**0**	**6**	**6**	**0**	**6**	

Literature review of rat hippocampus after MRI segmentation.

**Table 4:** Granule cell volume.

		**Schmitz et al. 2002**	**Hosseini-S et al. 2008**	**Our study 2013**
Imaging method		Histology	Histology	MRI
Animal	Species	Rat Long Evans	Rat Wistar	Rat Sprague-Dawley
	Age (weeks)	10	8	12
	Gender	Male rats	Male rats	Male rats
	Weight (g)	NA	200–220	300-400
**Volume in mm^3^**		**0.8 ±0.059(SEM**)	**1.7±0.03(SEM**)	**1.4± 0.2(SD**)

Literature review of granule cell layer volumes measurements after MRI segmentation.

### Effects of MRI contrast agent

**Figure 5:**
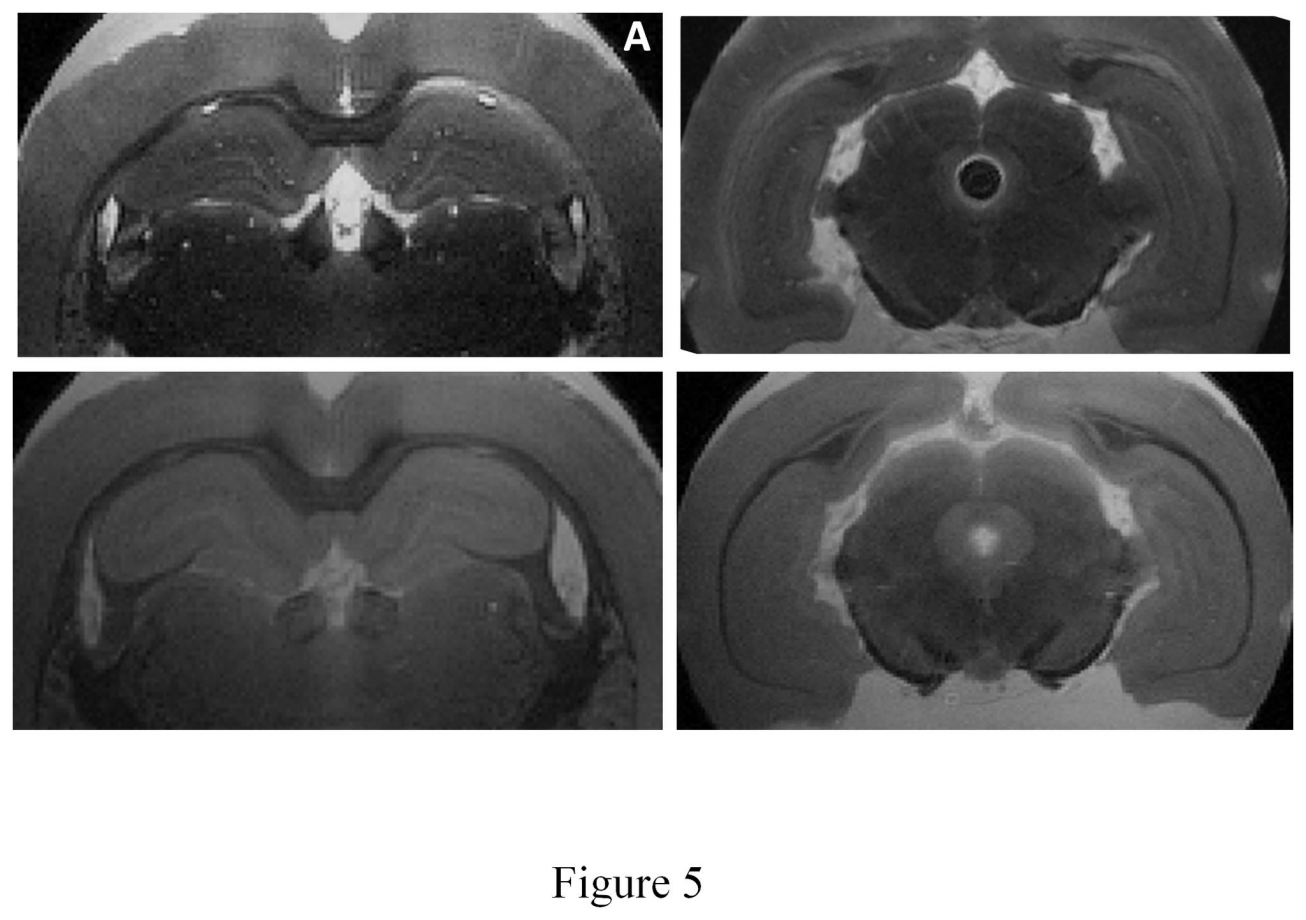
Hippocampal sub-layers visualization increased by MRI contrast agent. Comparison of MRI images without gadolinium (*below*) and after 70 min of passive staining (*above*) (A3) on dorsal hippocampus (*left*) and ventral hippocampus (*right*). The same number of layers can be seen with a better visualisation of the ventral hippocampus.

The acquisition time was decreased by 4 (3 h) after 70 min of passive staining (Table 2). The CNR appeared to be increased (*n*=1). The contrast between layers was visually increased even in the ventral part of the hippocampus (Figure 5).

## Discussion

The optimized conventional 3D T2 RARE sequence performed with the most commonly used surface coil on a 7T MRI resulted in a visual demonstration of hippocampal microstructure and calculation of thickness and volume layers. Contrary to previous studies, no complex post-processing, advanced DTI sequences, specific expensive coils, or high magnetic fields were required. We observed six rat hippocampal layers at 7T MRI that were qualitatively correlated to histological layers, whereas previous studies were performed at 9.4T and 7T MRI in mice [12,13,20,21]. Only one study performed at 7T in rats [22] with the aim of creating an MRI stereotaxic atlas has similarly reported six hippocampal layers at high resolution, but without measuring hippocampal volume or hippocampal layers volume. Additionally, the signal quality allowed us to segment one of the thinnest layers of the hippocampus, the granular cell layer. We have calculated its volume, in contrast to previous works where the volume of several layers was taken together or the volume of hippocampal area/regions included several partial layers taken together [23].

### T2 acquisition parameters

T2 contrast was chosen since it is widely used in research in small animals and is easy to compare to the literature. Indeed, the T1 contrast correlated to the magnetic field is decreased at high-field [24]. Without any contrast agent, and at high field, contrast is decreased because T1 differences smaller. Moreover T2 offers a better SNR in a high field but a lower SNR in a low field. We think that the better hippocampal layer depiction from T1 (voxel size =21.5 µm) compared to T2 weighted imaging (voxel size=43 µm) reported by Johnson et al. [20] was probably related to the difference in spatial resolution rather than the type of weighting. As the spatial resolution was improved, the thickness of the layers became more visible.

The spatial resolution or voxel size depends on the FOV, the size of the matrix and slice thickness reached up to 80 x 80 x 80 µm3, and allowed us to distinguish layers of up to 80 µm in thickness. To visually detect a layer, the voxel size has to be smaller than or equal to its thickness. Thus, the stratum granulare (500 µm thick) and the stratum radiatum (100 µm thick) are distinctly visible. Thinner layers such as the polymorphic layer are not visible.

Contrast parameters of TE and TR were used in order to differentiate the hippocampus from the other cerebral structures as distinctly as possible. The acquisition time, depending on TR, RARE Factor, and the NEX was decreased to its minimum, allowing one acquisition per night. Our unstained rat brain acquisition times (12.5 h) was much longer than those of other studies, ranging from 20 min to 3 h [1,4,10,11], which focused on hippocampus volumetric measurements and the demonstration of signal anomaly after brain lesion in rat models. Additionally, anisotropic voxels, lower spatial resolution, and thicker slices allowed shorter acquisition times, but no MRI microscopy as in our study.

Recent studies [12,13,25] allowed MR microscopy with shorter acquisition times, but their anisotropic voxels did not allow them to calculate layer volumes (partial volume effect). Their MRI was performed with high field strength not routinely available in preclinical research, and with the use of an expensive phase-array coil allowing them to further increase the SNR. MRI T2 sequencing was performed by Johnson et al. [22] in five rats at 7T with high-strength gradients (750 mT/m) and a 3D T2GR weighted sequence (TR: 50 ms;TE:8.3 ms;NEX:2; FOV: 40*20*20, Partial Fournier Acquisition Strategy with expanded dynamic range PFAS). Consequently, their voxel size was three times smaller than ours (25 µm). However, their acquisition time remained four times slower (13 hours) even though an active staining procedure was also performed.

The high SNR and CNR in our acquisition facilitated the delineation of the hippocampus, particularly the dorsal area, because of the surface coil. The ventral portion was less distinct due to the choice of surface coil, but was counterbalanced by the three-dimensional display of the hippocampus, particularly when layers were delineated.

### Hippocampal layers and settings

In our study, we observed that hippocampal layers with similar composition produced the same type of signal. Signals from the cellular layers such as the stratum granulare and the stratum pyramidal appear hyper-intense, and those of mixed layers composed of neural bodies, glia and dendrites (neuropil) appear iso -intense; this latter type of layer includes the stratum oriens, the stratum radiatum and the stratum lacunosum moleculare of the Ammon’s Horn and the stratum molecular layer of the dentate gyrus.

Cerebral structures largely composed with white matter like corpus callosum and fimbria appeared hypo-intense. Data found in the literature are not as linear as ours for the same type of sequence, the signals from the layers varying from one study to another (Table 5). This could be explained by the fact that the phenomena underlying T1 and T2 relaxation in these tissues are complex and difficult to predict. However, the hippocampal layers distinguished by Johnson et al. [22] in rats showed a similar type of signal except for the stratum lacunosum-moleculare, which appeared hypo-intense. In their study, the pyramidal layer was better delineated due to higher resolution (25 µm), but the polymorphic layer remained unobservable in both studies.

**Table 5:** Review of hippocampus MRI signals.

		**Wieshmann et al. 1999** [29] **6 layers**	**Yushkevitch et al. 2009** [30] **4 layers**	**Boretius et al. 2009 7 layers**	**Johnson 2012 6 layers**	**Our study 2013 6 layers**
Samples		Human Hippocampus samples *ex vivo*	Human Hippocampus samples *ex vivo*	Mice *in vivo*	Rat *ex vivo*	Rat *ex vivo*
Stratum oriens		Hyper	Hyper	Iso	Iso	Iso
Stratum pyramidale		Hyper	Hyper	Hypo	Hyper	Hyper
Stratum radiatum		Hypo	Hypo	Iso	Iso	Iso
SlmCA1		Hyper	Hypo	Hypo	Hypo	Iso
SmDG		Hyper	------	Iso	Iso	Iso
SgDG		Hypo	------	Hypo	Hyper	Hyper
PlDG		------	------	Hypo	------	------
**MRI/Weighting**		**7T/T^2^ SE**	**9.4 T/T^2^**	**9.4T/T2**	7T/ 3DT2*	**7T/T2**
MRI/Parameters	TR (msec)	3030	4000	4200	50	1700
	TE (msec)	60	26	82	8,3	63
	Flip Angle				60	
	Voxel size (µm^3^)	64x64x100	200x300x200	53x53x243	25x25x25	80x80x80
	AT (hours)		10	1	13	12.5

Comparison of layer signals in our study and in the literature and MRI parameters used (**SmDG**: stratum moleculare of the dentate gyrus, **SgDG**: stratum granulare of the dentate gyrus, **PlDG**: polymorphic layer of the dentate gyrus).

Histological sections performed from the same brain after MRI acquisition facilitated the delineation of MRI hippocampal layers and could be merged to better segment thick layers. However, it appears that the Cavalieri method (stereological method from histological brain sections) was less effective/than those of 11.4T MRI 3D RARE isotropic sequences (55 µm) in calculating the volume of selective cerebral structures in mice [23], so was not included in our study.

### MRI contrast agent

Passive staining with MRI contrast agent increased the CNR, particularly in the ventral area, as similarly reported by Johnson et al. [22], and decreased the acquisition times, as described in Dhenain’s study [17]. However, the dose of contrast agent must be carefully determined, as the signal was weakened with higher concentrations of gadolinium (data not shown) due to a large decrease in T1 relaxation time and T2 relaxation effects. Additionally, the relaxivity index of the contrast agent must be taken into account because of its ability to decrease T1 relaxation times, thus reinforcing the CNR. In contrast, manganese-ion enhancement allows better separation of cellular layers such as the granular layer, the pyramidal layer, and the polymorphic layer [25]. Manganese-ion enhancement could be tested to improve the separation of the pyramidal and polymorphic layers which are not perfectly delineated with the size of the voxel chosen in our study. However manganese ions induce cerebral toxicity [26], so we preferred Dotarem regarding the next step of this project where, similar sequences will be tested ex-vivo in order to visualize and calculate hippocampal layers in a model of vestibulo-deficient rats where hippocampus volume might be decreased by the vestibular lesion. Additionally, Dotarem decreases T1 relaxation time in all tissues; consequently it decreases the acquisition time of the whole tissue sample volume by four, regardless of the type of tissue. Finally, passive staining with an MRI contrast agent such as Dotarem significantly decreased the acquisition time and increased the contrast between layers, extending acquisition potential to large groups of samples.

### Volume measurements

Manual segmentation of the hippocampus was facilitated by three-dimensional acquisition and the choice of isotropic voxels. We switched between each axis when in doubt during the outlining procedure of the hippocampus. The hippocampus volumes reported here with our sequence were similar to those in the literature. Though it appears imprecise in the figure, the segmentation of the granule cell layer was performed point by point, and the volumes calculated were similar to those obtained from histology [8,27]. To the best of our knowledge, no hippocampal layer measurements have been reported using MRI acquisition. In a recent study by Deweurwaerdere et al. [28] the thickness of the CA1 region was calculated from manganese T1 MRI acquisition (MEMRI) and was compared to neuronal loss calculated from histological sections in a rat model of epileptic seizures. However their spatial resolution did not allow them to separate or calculate hippocampal layers.

### Technical limitations

#### Surface coil

The surface coil used in this study, supplied with the equipment and by Bruker, was more sensitive but less homogeneous than a solenoid RF coil would have been. Therefore, the SNR decreased progressively with the distance to the antenna and explains why the ventral portion of the hippocampus was less clearly delineated. Phase-array coils composed of four elements might increase the SNR and would thus improve the delineation of the rat hippocampus.

#### Strength of magnetic field and gradients

The specific tools used to differentiate hippocampal layers are summarized in Table 6, showing that spatial resolution was higher than that used in our study and that one additional hippocampal layers were reported by these authors.

**Table 6:** MRI tools review.

	**Beneviste et al. 2000/2002 Mice ex vivo**	**Johnson et al. 2007 Mice ex vivo**	**Badea et al 2007 Sharief et al. 2008 Mice ex vivo**	**Boretius et al 2009 Mice in vivo**	**Deweur- waerde 2013 Rat in vivo**	**Johnson et al 2012 Rat ex vivo**	**Kamsu et al 2013 Rat ex vivo**
Magnetic Field Strength (Tesla)	9,4	9.4	9,4	9.4	4,7	7	7
**Magnetic field strength (Tesla)**	**NA**	**950**	**850**	**950**	**NA**	**750**	**300**
Radio-frequency coil	Solenoid coil	Solenoid coil	Solenoid coil	Phase-array coil	Surface Coil	Solenoid coil	Surface coil
**Pre or post processing**	**--------**	**PFAS**	**MEFIC**	**----------**	**No**	**PFAS**	**No**
Spatial resolution (µm^3^)	39X39X156	43x43x43	43x43x43	53x53x243	90x130x390	25x25x25	80x80x80
**Staining**	**Active**	**Active**	**Active**	**No**	**Manganese**	**Active**	**No**

Specific tools used to increase SNR and CNR without losing spatial resolution

(T2 contrast except for Deweurwaerdere et al, T1 contrast), (**MEFIC**: multi-echo frequency domain image, **PFAS**: partial Fournier acquisition strategy with expanded dynamic range).

## Conclusions

In this study we address the question of whether the hippocampal microstructure can be visually detected with a conventional MRI sequence, a surface coil, and a standard research magnetic field at 7T whose use is widespread in research centres. We optimized a new RARE sequence of 12.5 hours, allowing the differentiation of six layers of rat hippocampus. Our sequence allowed us to calculate, using MRI, not only the total hippocampal volume, but also the volume of a very thin cellular layer, the granule cell layer (1 mm^3^), which to our knowledge has never been reported before. Additionally, we have improved the MRI volume measurements of rat hippocampus by using isotropic voxels. We also present the findings of a passive staining protocol, which allowed us to decrease the scan time by 4 and increase the CNR. Spatial resolution might be improved on a 7T MRI by combining our 3D RARE T2 sequence with a higher gradient strength and using a specific coil and partial Fourier strategy with extended dynamic range.
